# The influence of salivary contamination on the shear bond strength of two newer generation dentin bonding agents - An *in vitro* study

**DOI:** 10.4103/0972-0707.45252

**Published:** 2008

**Authors:** Mithra N Hegde, Priyadarshini Hegde, Shibani K Shetty

**Affiliations:** Department of Conservative Dentistry and Endodontics, A. B. Shetty Memorial Institute of Dental Sciences, Deralakatte, Mangalore, Karnataka, India

**Keywords:** Bond strength, dentin bonding agents, salivary contamination

## Abstract

**Background and Objectives:**

To investigate whether salivary contamination during various stages of the bonding procedures of Xeno III and Clearfil SE Bond influences shear bond strength.

**Materials and Methods:**

The occlusal surfaces of thirty six maxillary premolar teeth were ground and divided into two groups containing eighteen specimens each, which was subdivided into three sub groups: Group I - Xeno III, Group II - Clearfil SE Bond, Subgroup A - Uncontaminated (control), Subgroup B - Contaminated with saliva before application and light curing, Subgroup C - Contaminated with saliva after light curing. Composite resin Filtek Z350 was packed using Teflon mold cured and subjected to shear bond strength analysis using universal Instron machine.

**Results:**

Statistical analysis was made using One-way ANOVA and Tukeys HSD test. Clearfil SE Bond showed very high statistically significant reduction in the bond strength, when salivary contamination took place after light curing; whereas, Xeno III showed very high statistically significant reduction when salivary contamination took place before application and light curing.

**Conclusion:**

Clearfil SE Bond showed more tolerance to salivary contamination of dentin and higher shear bond strength value, when compared to Xeno III.

## INTRODUCTION

Research for improved adhesive restorative material has been a subject of considerable research in recent years.[1] Clinically, there have been many factors which affect adhesion and retention of resin containing restorative materials such as moisture, gingival fluid, blood, handpiece oil and, in particular, saliva which can affect the bond strength.[1]

Earlier studies by Johnson *et al.* have suggested that the clinical success of resin bonding systems to enamel and dentin could be jeopardized by contamination with saliva.[2] *In vitro* research by Fritz *et al.* has also indicated that salivary contamination of the cured adhesive layer has a detrimental effect on bond strength values.[3] Dentin adhesion is extremely complex when compared with enamel bonding and micromechanical resin adhesion to dentin differs fundamentally from the relative simple interlocking of bonding agents with enamel.

Recently developed adhesive systems such as the one-step self-etching adhesive and two-step self-etching adhesive have simplified the bonding procedures by etching the tooth surface and simultaneously preparing it for adhesion. Even though there is reduced risk of saliva contamination, it may sometimes be impossible to maintain an uncontaminated operative field, resulting in reduced bond strength.[4] Very few studies have investigated the effect of saliva contamination on the bonding properties of self-etching adhesive systems. The results of many studies are not in agreement with each other.

The aim of the present study is to evaluate the influence of salivary contamination during the various stages of bonding procedures on the shear bond strength of newer generation dentin bonding agents like one-step self-etch adhesives and two-step self-etch adhesives.

## MATERIALS AND METHODS

Thirty six freshly extracted non-carious human maxillary premolars were selected for this study. All the collected teeth were cleared of blood and saliva and stored in buffered isotonic saline solution. The occlusal surfaces of the teeth were ground on water cooled trimming wheel, to prepare flat dentin surfaces at a depth of 1.5 mm from the cuspal tip of the tooth. They were randomly divided into two groups, based on the dentin bonding agent used with the eighteen specimens in each group, as follows:

**Group I** - Xeno III

**Group II** - Clearfil SE Bond

The operative procedure followed for the bonding agents are presented in Table 1. Each group was further randomly subdivided into three subgroups, with six specimens in each group. In subgroup B and Subgroup C, the specimens were contaminated with fresh whole saliva, which was painted for 20 seconds using a disposable brush saliva, at different stages of bonding [Table 2].

**Table 1:** Operative procedures followed for the bonding agents - Xeno III and clearfil SE bond

Bonding agent/Composition	Bonding agent treatment	Restoration
**Group I** - Xeno III (Dentsly/Caulk) Liquid A: HEMA, Ethanol, water, highly dispersed silicondioxide, BHT Liquid B: phosphoric acid, monofluorophosphazene, modified methacrylate resins, UDMA, BHT, Camphoroquinone, ethyl-4-dimethyl aminobenzoate.	Equal amounts of liquid A and liquid B was mixed in a clean mixing well for approximately five seconds. Two coats were applied to thoroughly wet all dentinal surface and left undisturbed for at least 20 seconds. Adhesive was spread uniformly using a gentle stream of air, until there was no more flow of adhesive, to ensure proper removal of the solvent. Light curing was done for 20 seconds.	Z350(3M) was placed in increments using a Teflon mold measuring 2 mm × 2 mm and cured layer by layer for 40 seconds.
**Group II** - Clearfil SE Bond (Kurray) SE Primer: MDP, HEMA, NN-diethanol, p-toluidine, hydrophilic dimethacrylates, and water SE Bond: MDP, Bis GMA, HEMA, Hydrophobic dimethacrylate, CQ, NN- Diethanol, p toluidine with silanated colloidal silica	The self-etching primer was applied and allowed to act for 20 seconds, was briefly air dried. Two coats of the bonding agent were applied and spread with a light stream of air. Light curing was done for 20 seconds.	Z350(3M) was placed in increments using Teflon mold measuring 2 mm × 2 mm and cured layer by layer for 40 seconds.

**Table 2:** Operative procedures for the subgroups of the bonding agents - Xeno III and clearfil SE bond

Subgroups	Stage of bonding at which the salivary contamination was done
Subgroup A	Uncontaminated specimens (control).
Subgroup B	Specimens contaminated with saliva for 20 seconds, followed by application of the bonding agent.
Subgroup C	Specimens contaminated with saliva for 20 seconds, after light curing of the bonding agent

**Subgroup A** - Uncontaminated specimens (control)

**Subgroup B** - Specimens contaminated with saliva for 20 seconds, followed by application of bonding agent

**Subgroup C** - Specimens contaminated with saliva for 20 seconds, after light curing of the bonding agent

The prepared specimens were mounted on metal cylinders using dental stone in such a way that the flat dentin surface was 5 mm from the metal cylinder mold. The specimens were kept in a distilled water bath for 24 hours at 37°C. All 36 specimens were transferred to the universal Instron machine individually and subjected to shear bond strength analysis at a crosshead speed of 1.0 mm/minute, in the Department of Metallurgy, National Institute of Technology, Surathkal, Karnataka.

## RESULTS

The data obtained in the present study was subjected to statistical analysis using One-way anova, followed by Tukeys HSD test.

When a comparison of the shear bond strength of the uncontaminated specimens with the contaminated specimens in each group was made using One-way ANOVA [Table 3], it showed very high statistical significance.

**Table 3:** Mean and standard deviation values for shear bond strength using one-way ANOVA

Group	Subgroup	n	Mean	Std. deviation	F	*P*
	Subgroup A	6	25.0917	0.66947		
Group I	Subgroup B	6	20.0492	0.6084	87.114	0.0001 vhs
	Subgroup C	6	23.9075	0.78629		
	Subgroup A	6	26.4175	0.36043		
Group II	Subgroup B	6	25.7892	0.35831	22.925	0.001 vhs
	Subgroup C	6	25.0783	0.30688		

F- One-way ANOVA test

When comparisons of the shear bond strength of subgroups within the group was done using Tukeys HSD test [Table 4]. The results were as follows: Group I (Xeno III) - Control versus subgroup B showed very high statistical significance. Group II (Clearfil SE Bond) - Control versus subgroup B showed no statistical significance. Control versus subgroup C showed statistical significance.

**Table 4:** Comparison of shear bond strength values of the subgroups within the group in group I and group II using Tukeys HSD test

Group	Control	Experimental	Mean difference	*P*
Group I	Subgroup A	Subgroup B	5.0425	0.001 vhs
		Subgroup C	1.1842	0.025 sig
Group II	Subgroup A	Subgroup B	0.6283	0.06 ns
		Subgroup C	1.3392	0.032 sig

t-Tukeys HSD *P*< 0.05 - Significant (sig) *P*< 0.01 - Highly significant(hs), *P*< 0.001 - Very highly significant (vhs) *P*> 0.05 - Not significant (ns)

When an intergroup comparison of shear bond strength values between the subgroups of the two groups were made using Tukeys HSD test [Table 5], the values of the control group showed high statistical significance. When compared with the subgroup B values, it showed very high statistical significance. In subgroup C, Group I versus Group II showed high statistical significance. Group I (Xeno III) showed a decrease in shear bond strength values, to a greater extent, when compared with Group II (Clearfil SE Bond), which was more stable.

**Table 5:** Intergroup comparison of shear bond strength values between the subgroups of group I and group II using Tukeys HSD test

Subgroup	Group	*n*	Mean	Standard deviation	t
Subgroup A	Group I	6	25.0917	0.66947	4.271
	Group II	6	26.4175	0.36043	*P* = 0.002 hs
Subgroup B	Group I	6	20.0492	0.6084	19.913
	Group II	6	25.7892	0.35831	*P* = 0.001 vhs
Subgroup C	Group I	6	23.9075	0.78629	3.391
	Group II	6	25.0783	0.30688	*P* = 0.007 hs

t-Tukeys HSD *P*< 0.05 - Significant (sig) *P*< 0.01 - Highly significant (hs), *P*< 0.001 - Very highly significant (vhs) *P*> 0.05 - Not significant (ns)

## DISCUSSION

One of the clinical technique sensitive factors that can affect adhesion and retention of resin restorative materials is contamination of the field with saliva.[5] In the present study, Xeno III, a one-step self-etch adhesive, demonstrated fairly good bond strength values with dentin. Van Meerbeek *et al*. attributed the fairly good bond strength values obtained with Xeno III to it being an intermediary strong self-etch adhesive with an acidic pH of 1.4, which results in better micromechanical interlocking to enamel and dentin, compared to mild self-etch adhesives.[6]

Xeno III showed comparatively lower bond strength values when dentin was contaminated with saliva, before application and light curing of the adhesive. This is in agreement with the results of a study done by Yoo *et al.* in which SEM also showed less demineralization and less infiltration of the adhesive, which may have resulted in the decrease in bond strength values.[7] Park *et al.* stated that the presence of saliva may result in the dilution of adhesive resulting in a weak hybrid layer.[4] Studies done by Pereira *et al.* showed that dilution of water based system results in a reduction in the degree of monomer conversion and a reduction in the bond strength. Xeno III contains ethanol, water and a phosphate ester.

The present study showed slight reduction in the shear bond strength values when contaminated with saliva after light curing of the adhesive, which was statistically significant. This could be due to the adsorption of glycoproteins onto the poorly polymerized adhesive surface, which results in oxygen inhibition.[7]

The results of the present study is in agreement with the result of an earlier study done by Fritz *et al*. using one-step adhesive, which concluded that in one bottle adhesive system, any contamination of the already cured adhesive layer seriously compromises the bond strength.[3]

In the present study, it was observed that Clearfil SE Bond showed good degree of tolerance to salivary contamination, when compared with Xeno III. Summitt James attributed the high bond strength values obtained with Clearfil SE Bond to the presence of methacryloyloxydecyl dihydrogen phosphate (MDP), which improved adhesion to enamel and dentin, and has a long hydrophobic and short hydrophilic group that improves wetting of the hydrophilic dentin surface.[8] Being a mild acid, it causes only superficial demineralization to occur partially, keeping the residual hydroxyapatite still attached to the collagen, which may serve as a receptor for additional chemical bonding.[6] Studies by Yoshida *et al.* showed that Methacryloyloxydecyl dihydrogen phosphate (MDP) has a chemical bonding potential to calcium of residual hydroxyapatite.[9] It also protects the collagen against hydrolysis and, thus, early degradation of the bond.[10]

Kalla El *et al*. hypothesized that hydrophilic solutions, in particular acetone or ethanol based products, may displace or diffuse through a saliva film to reach the underlying hydroxyapatite or collagen as a condition for firm bonding after polymerization.[11] This explains the high bond strength values obtained in the presence of contamination by Clearfil SE, which contains NN-Diethanol in the composition of the SE Primer as well as the SE Bond. The result is in accordance with an earlier study done by Park *et al.*, which showed that Clearfil SE Bond tolerated salivary contamination, except when the contamination occurred after primer application.[4]

The decrease in shear bond strength was statistically significant, when contamination occurred following light curing of the adhesive. This is in agreement with the results of the study done by Fritz *et al.*[3] using one bottle adhesive systems, which concluded that in one bottle adhesive system, any contamination of the already cured adhesive layer seriously compromises the bond strength. They put forward mainly three different hypotheses for the reduction in shear bond strength values:

1. Adsorption of glycoproteins to the poorly polymerized adhesive surface, thus preventing adequate co-polymerization.
2. Compromise of the co-polymerization with the subsequent resin layer, by removal of the oxygen inhibited un-polymerized surface layer, during rinsing and drying.
3. Insufficient filling of the collagen mesh with resin.[3]

Davidson *et al.* postulated that minimum bond strength of 17-20 MPa to enamel and dentin is needed to resist contraction forces of resin composite materials.[12] In the present study, both the self-etching adhesives showed optimal bond strength values greater than 20 MPa for both uncontaminated and contaminated dentin.

## CONCLUSION

Within the limitations of the *in vitro* study, the following conclusions were drawn:

- Salivary contamination of dentin occurring during the various stages of bonding had a detrimental effect on the shear bond strength of both Clearfil SE Bond and Xeno III. Clearfil SE Bond demonstrated good degree of tolerance to salivary contamination.
- Xeno III showed significant reduction in bond strength, when salivary contamination of dentin took place before application and curing the adhesive. Whereas, Clearfil SE Bond showed significant reduction in bond strength, when salivary contamination took place after curing the adhesive.
- The newer generation self-etching adhesive systems showed optimal bond strength values in both uncontaminated and contaminated dentin for successful retention of resin restoration
